# Intrahepatic cholestasis of pregnancy

**DOI:** 10.1186/1750-1172-2-26

**Published:** 2007-05-29

**Authors:** Thomas Pusl, Ulrich Beuers

**Affiliations:** 1 Department of Medicine II, Klinikum Grosshadern, University of Munich, Munich, Germany; 2 Department of Gastroenterology & Hepatology, AMC, University of Amsterdam, The Netherlands

## Abstract

Intrahepatic cholestasis of pregnancy (ICP) is a cholestatic disorder characterized by (i) pruritus with onset in the second or third trimester of pregnancy, (ii) elevated serum aminotransferases and bile acid levels, and (iii) spontaneous relief of signs and symptoms within two to three weeks after delivery. ICP is observed in 0.4–1% of pregnancies in most areas of Central and Western Europe and North America, while in Chile and Bolivia as well as Scandinavia and the Baltic states roughly 5–15% and 1–2%, respectively, of pregnancies are associated with ICP. Genetic and hormonal factors, but also environmental factors may contribute to the pathogenesis of ICP. Intrahepatic cholestasis of pregnancy increases the risk of preterm delivery (19–60%), meconium staining of amniotic fluid (27%), fetal bradycardia (14%), fetal distress (22–41%), and fetal loss (0.4–4.1%), particularly when associated with fasting serum bile acid levels > 40 **μ**mol/L. The hydrophilic bile acid ursodeoxycholic acid (10–20 mg/kg/d) is today regarded as the first line treatment for intrahepatic cholestasis of pregnancy. Delivery has been recommended in the 38^th ^week when lung maturity has been established.

## Disease name and synonyms

Intrahepatic cholestasis of pregnancy

Obstetric cholestasis Recurrent jaundice of pregnancy

Pruritus gravidarum

Icterus gravidarum

Idiopathic jaundice of pregnancy

## Definition

Intrahepatic cholestasis of pregnancy (ICP) is a cholestatic disorder characterized by pruritus, elevated serum aminotransferases and bile acid levels with onset in the second or third trimester of pregnancy, and spontaneous relief of signs and symptoms within two to three weeks after delivery [1,2].

In the first description of ICP in 1883, Ahlfeld described maternal pruritus and jaundice in the last trimester of pregnancy disappearing after delivery [3].

## Epidemiology

ICP has been observed in almost all ethnic groups, but there is relevant geographical variation in the incidence of ICP [1,4]. The incidence of ICP was highest in Bolivia and Chile several decades ago (up to 14% of all pregnancies before 1975), particularly among the Araucanos Indians of Chile (27.6%) and the Aimara Indians of Bolivia (13.8%) [5-8], but has considerably decreased in these countries more recently to less than 2% of all births today [7]. In Scandinavian and Baltic countries, ICP occurs in up to 2% of all pregnancies, while in other regions of Europe, Asia, North America and Australia the reported incidence is less than 1% [1,7,9]. While the incidence has recently decreased in the high-incidence regions, it increased in low-incidence areas, possibly reflecting the raising awareness of the disease [4,7]. ICP is more common in the winter months in Chile and Scandinavia [10,11], and in twin and multiple pregnancies [12].

## Clinical manifestations and biochemical tests

Pruritus is the primary clinical symptom of ICP. Pruritus may be mild and tolerable for some patients, but may also be very severe and disabling. It may considerably impair the patient's quality of life causing sleep deprivation, psychological suffering and even suicidal thoughts. Pruritus is most severe in the evening, with a predilection for the palms of the hands and soles of the feet, and is not associated with any specific skin lesions. It usually presents in the third trimester, after 30 weeks of gestation, but rare cases developing as early as 6 to 10 weeks have been described [9,13,14]. Mild jaundice with serum levels of conjugated bilirubin only moderately elevated occurs in 10 to 15% of cases [9,15]. Jaundice typically develops 1–4 weeks after the onset of pruritus, but occasionally can be the initial symptom [16,17]. Subclinical steatorrhea may be seen along with fat malabsorption, which may lead to vitamin K deficiency resulting in a prolonged prothrombin time and postpartum hemorrhage [18,19]. The incidence of gallstone formation and cholecystitis (rate ratio 3.7) is higher in women with a history of ICP (as well as in their first degree relatives) than in the normal population [20]. Abdominal pain, malaise and other constitutional symptoms are uncommon.

The main biochemical alterations are elevations of serum bile acids and aminotransferase activities [21]. Serum total bile acid levels may increase 10–100 times above the normal range and higher fetal complications rates were observed with maternal fasting bile acid levels exceeding 40 **μ**mol/L [22-25]. In ICP, cholic acid is raised more than chenodeoxcholic acid with an increase in the usual molar ratio of serum cholic to chenochenodeoxcholic acid, whereas the ratio of glycine- to taurine-conjugated bile acids decreases [13,26-28]. Of interest, a subgroup of asymptomatic healthy pregnant women with total serum bile acids above the upper normal limit of 11 **μ**M in late gestation and normal serum liver tests has recently been defined as asymptomatic hypercholanemia of pregnancy (AHP) [29,30]. The serum bile acid composition of women with ICP revealed a shift towards a more hydrophobic pattern with higher levels of lithocholic acid and unconjugated serum bile acids, suggesting that these alterations could be additional useful parameters in the differential diagnosis of ICP and AHP [30]. Serum aminotransferases are also elevated 2–10-fold above normal in 20–60% of patients with pruritus, and may exceed 1000 U/L in exceptional cases [1,9,10,16,31]. Serum aminotransferases may allow better to follow patients with ICP after start of UDCA treatment than total fasting serum bile acid levels which initially increase due to an increase in ursodeoxycholic acid (UDCA) serum levels. Serum concentrations of gamma glutamyl transpeptidase are normal or modestly elevated in half of the patients, reaching up to four times the upper normal limits [9,13]. Serum levels of alkaline phosphatase may rise up to 7–10 times normal, but are difficult to interpret due to elevation of the placenta isoenzyme [32].

### Maternal outcome

Maternal prognosis is good and symptoms resolve rapidly after delivery, accompanied by normalization of serum liver tests [28]. Persistent abnormalities should prompt reconsideration of other underlying chronic liver diseases like primary biliary cirrhosis, primary sclerosing cholangitis, or chronic hepatitis C which all may be associated with development of pruritus during late pregnancy. ICP recurs during subsequent pregnancies in 45–70% with varying severity of recurrent episodes [9].

### Fetal outcome

ICP increases the risk of preterm delivery (up to 19–60%) [9,33,34], meconium staining of amniotic fluid (up to 27%) [35], fetal bradycardia (up to 14%) [35], fetal distress (up to 22–41%) [11,33,36], and fetal loss (up to 0.4–4.1%) [33,37,38], particularly when associated with fasting serum bile acid levels > 40 **μ**mol/L [25]. Fasting serum bile acid levels and severity of pruritus at diagnosis were independent predictors of preterm delivery in a recent analysis (Kondrackiene *et al*., submitted). Interestingly, most recent data show a marked decrease in fetal complications [25,39-42] possibly in part due to greater awareness for the disease, closer follow-up and experienced management in specialized centers. Autopsies of the stillborns show signs of acute anoxia with serosal and pulmonary petechial bleeding without intrauterine growth retardation [18,35,43]. The pathogenesis of the fetal complications is still poorly understood, although a role for bile acids or toxic metabolites of bile acids has been suggested [4]. Bile acids were shown to induce contraction of the chorionic veins of the placenta, and myometrial sensitivity of healthy women to oxytocin was increased after incubation with cholic acid [44,45]. The infusion of cholic acid in fetal lambs stimulates colonic motility increasing the incidence of meconium passage [46]. In a prospective study of ICP in patients with bile acid levels > 40 **μ**mol/L the frequency of meconium passage was 44% compared to 22% in a group with only mild ICP [25].

## Etiology and pathogenesis

The etiology of ICP is not completely understood and is still under discussion. Genetic and hormonal factors, but also environmental factors may contribute to the pathogenesis of ICP [1]. Familial clustering, ethnic and geographic variation, and the high rate of reoccurrence in subsequent pregnancies support a genetic predisposition for ICP [1,47]. Mutations in the hepatocellular phospholipid transporter, ABCB4 (MDR3), that mediates secretion of the major human phospholipid, phosphatidylcholine (lecithine) into bile, have been estimated to account for up to 15% of all ICP cases [21,48,49]. Available molecular genetic analysis suggests that other major ABC transporters of liver cells, the bile salt export pump (BSEP), ABCB11, and the aminophospholipid transporter (FIC1), ATP8B1, are less likely to be implicated in the pathogenesis of ICP [21,50-52].

Clinical evidence supports an etiologic role for estrogens in the initiation of ICP [1,53,54]. ICP most commonly occurs in the last trimester, when estrogen levels reach their maximum. ICP has been associated with twin and triplet pregnancies with higher estrogen levels than single gestations. Finally, estrogen oral contraceptive use among women with a personal or family history of ICP could result in clinical features of ICP particularly when former high-dose preparations were used [1,4,32]. Progesterone and associated metabolites may also be involved in the pathogenesis of ICP. Patients with ICP have significantly increased plasma levels of mono- or disulfated progesterone metabolites and an increased ratio of 3**α**-hydroxylated steroids to 3**β**-hydroylated steroids [47,55,56]. Some estrogens, in particular 17**β**-D-glucuronide, and sulfated progesterone metabolites have been shown to cause cholestasis [57], but the molecular mechanism is still under discussion. Impairment of the function of major hepatocellular ABC transporters like the bile salt export pump (BSEP), ABCB11, or the phospholipid transporter, ABCB4 (MDR3), by high levels of estrogen glucuronides and progesterone, respectively, at the posttranscriptional level has been demonstrated *in vitro *[58-60]. In addition, estrogens impaired basolateral as well as canalicular bile acid transporter expression of liver cells *in vitro *by transcriptional mechanisms [61].

Thus, mutations in genes encoding hepatobiliary transport proteins as well as abnormal metabolites impairing hepatobiliary carriers may be involved in the pathogenesis of ICP.

The seasonal variation, the incomplete recurrence in subsequent pregnancies, as well as the decrease in the prevalence of ICP in high-incidence regions in association with improved nutritional supply suggest that exogenous factors such as nutritional factors like selenium deficiency may contribute to ICP [62,63].

## Diagnosis

The diagnosis of ICP is based on (i) pruritus of cholestasis, (ii) elevated fasting serum bile acids > 10 **μ**mol/L (and elevated serum transaminases), (iii) spontaneous relief of signs and symptoms within two to three weeks after delivery and (iv) absence of other diseases that cause pruritus and jaundice. Liver biopsy is not necessary for the diagnosis and histopathology is not diagnostic, showing centrilobular cholestasis without inflammation and bile plugs in hepatocytes and caniculi without bile duct dilatation or injury [16,64].

## Differential diagnosis

A number of other disorders may erroneously be interpreted as ICP during pregnancy, making exclusion of diseases associated with pruritus without cholestasis or with pruritus with cholestasis important. The differential diagnosis of ICP with pruritus (and elevation of serum bile acid levels) only includes skin diseases and specific dermatoses of pregnancy, allergic reactions, renal pruritus and hematological disorders such as Hodgkin's disease and polycythemia rubra vera. ICP with elevation of serum liver tests should be distinguished in the late 1^st ^trimester from hyperemesis gravidarum, in the 3^rd ^trimester from acute fatty liver of pregnancy, HELLP (a syndrome characterized by**H**emolysis, **E**levated **L**iver enzyme levels and a **L**ow **P**latelet count), pre-eclampsia, eclampsia, and in all trimesters from viral hepatitis, alcoholic hepatitis, drug-induced hepatitis, biliary obstruction, hyperbilirubinemic states, primary biliary cirrhosis and primary sclerosing cholangitis [13,47].

## Management

### Pharmacologic treatment

The primary objective of pharmacologic treatment in ICP is to alleviate maternal symptoms and improve fetal outcome. Antihistamines, benzodiazepines, phenobarbital, opioid antagonists, dexamethasone, epomediol, S-adenosyl-L-methionine and cholestyramine have been used but not introduced into clinical practice because of limited efficacy and/or tolerability [1,32,39].

Currently, the hydrophilic bile acid ursodeoxycholic acid (UDCA) is the most effective treatment for ICP. In an open, randomized, parallel group study, 84 symptomatic patients with ICP were randomized to either UDCA, 8–10 mg/kg body weight per day, or cholestyramine, 8 g per day, for 14 days. Relief of pruritus was significantly more pronounced in the UDCA group and serum alanine and aspartate aminotransferase activities and endogenous serum bile acid levels were more effectively lowered after UDCA therapy. In addition, delivery occurred closer to term in patients treated with UDCA [40]. A second double-blind, placebo-controlled trial comparing UDCA (1 g per day for three weeks) and dexamethasone (12 mg per day for one week) in 130 women with ICP demonstrated significant improvement of serum alanine aminotransferase and bilirubin levels irrespective of disease severity in the UDCA group only and significant improvement of pruritus in the subgroup of women in the UDCA group with bile acid levels = 40 **μ**mol/L at study entrance. In contrast, dexamethasone did not alleviate pruritus, and serum bile acids and bilirubin were less effectively reduced compared to the UDCA group. However, no differences in fetal complications were detected, possibly due to the fact that fetal complications were less frequently observed in the whole study population than in previous studies [39]. In a retrospective, non-randomized analysis of a 12-year observation period, 32 patients with ICP were treated with UDCA (15 mg/kg/day) for three or more weeks before delivery and compared with 16 historical untreated controls with similar clinical and biochemical characteristics. UDCA treatment significantly improved pruritus intensity and some biochemical markers of ICP and resulted in a higher proportion of deliveries at term with a higher birth weight compared with historical controls [21,41]. A recent randomized prospective comparative study of UDCA (750 mg per day) and S-adenosyl-L-methionine (1000 mg per day intravenously) of 78 patients with ICP suggested that both regimens improved pruritus, but the combined therapy had no additive effect on pruritus as compared to UDCA monotherapy [42]. UDCA seems to be well tolerated by pregnant women and no adverse effects in mothers or newborns have been observed [39-42]. The mechanisms underlying the beneficial effects of UDCA in ICP are not entirely clear. UDCA has been shown to improve impaired hepatocellular secretion by mainly posttranscriptional stimulation of canalicular expression of key transport proteins like the conjugate export pump, MRP2 (ABCC2), or the bile salt export pump, BSEP (ABCB11) [65,66]. In particular, targeting and insertion of these transporter proteins into the canalicular membrane by UDCA conjugates has been demonstrated in experimental models of cholestasis, which led to enhanced elimination of bile acid metabolites and other organic anions as well as steroid mono- and disulfates [65,66]. This mechanism might be crucial for the understanding of the beneficial effect of UDCA in ICP.

In addition to effects of UDCA on the maternal liver, UDCA restores the impaired maternal-placental bile acid transport across the trophoblast. This could be mediated by enhanced expression of plasma membrane transporters involved in the excretory role of the placenta and would prevent structural alterations of the trophoblast induced by maternal cholestasis [15,21].

## Delivery

Since conventional antepartum testing does not reliably predict fetal mortality and induction of labor in the 38^th ^week of gestation has been observed to reduce fetal risk, delivery has been recommended in the 37^th ^to 38^th ^week in ICP. In most severe cases (before the era of UDCA treatment), delivery has been initiated even at 36 weeks gestation as soon as lung maturity had been established [28,34,67]. As clearly stated by the *UK Guideline for Obstetric Cholestasis 2006*, "there are insufficient data to support or refute the popular practice of 'early' (37 weeks of gestation) induction of labour aimed at reducing late stillbirth. The timing and risks of delivery should, therefore, be discussed on an individual basis."
